# Novel Plasminogen Activator Inhibitor-1 Inhibitors Prevent Diabetic Kidney Injury in a Mouse Model

**DOI:** 10.1371/journal.pone.0157012

**Published:** 2016-06-03

**Authors:** Bo Yeong Jeong, Md Jamal Uddin, Jong Hee Park, Jung Hwa Lee, Hi Bahl Lee, Toshio Miyata, Hunjoo Ha

**Affiliations:** 1 Graduate School of Pharmaceutical Sciences, College of Pharmacy, Ewha Womans University, 52 Ewhayeodae-gil, Seodaemun-gu, Seoul, 120–750, Korea; 2 Kim’s Clinic and Dialysis Unit, Myrang, Korea; 3 Center for Translational and Advanced Research, Tohoku University Graduate School of Medicine, Sendai, Japan; Graduate School of Medicine, University of the Ryukyus, JAPAN

## Abstract

Diabetic nephropathy is the leading cause of end-stage renal disease worldwide, but no effective therapeutic strategy is available. Because plasminogen activator inhibitor-1 (PAI-1) is increasingly recognized as a key factor in extracellular matrix (ECM) accumulation in diabetic nephropathy, this study examined the renoprotective effects of TM5275 and TM5441, two novel orally active PAI-1 inhibitors that do not trigger bleeding episodes, in streptozotocin (STZ)-induced diabetic mice. TM5275 (50 mg/kg) and TM5441 (10 mg/kg) were administered orally for 16 weeks to STZ-induced diabetic and age-matched control mice. Relative to the control mice, the diabetic mice showed significantly increased (p < 0.05) plasma glucose and creatinine levels, urinary albumin excretion, kidney-to-bodyweight ratios, glomerular volume, and fractional mesangial area. Markers of fibrosis and inflammation along with PAI-1 were also upregulated in the kidney of diabetic mice, and treatment with TM5275 and TM5441 effectively inhibited albuminuria, mesangial expansion, ECM accumulation, and macrophage infiltration in diabetic kidneys. Furthermore, in mouse proximal tubular epithelial (mProx24) cells, both TM5275 and TM5441 effectively inhibited PAI-1-induced mRNA expression of fibrosis and inflammation markers and also reversed PAI-1-induced inhibition of plasmin activity, which confirmed the efficacy of the TM compounds as PAI-1 inhibitors. These data suggest that TM compounds could be used to prevent diabetic kidney injury.

## Introduction

Diabetic kidney disease is the leading cause of end-stage renal disease worldwide and an independent risk factor for cardiovascular morbidity and mortality [1]. Current therapy including tight control of blood glucose and blood pressure and inhibition of angiotensin might delay but does not stop the development and progression of kidney injury in diabetes [2]. Therefore, new and comparatively more effective therapeutic measures for diabetic nephropathy are essential.

Diabetic kidney injury is characterized by albuminuria, a reduced glomerular filtration rate, and excessive extracellular matrix (ECM) deposition, which leads to glomerular mesangial expansion and tubulointerstitial fibrosis [3–5]. ECM accumulation is the net result of the balance between ECM synthesis and degradation, and ECM degradation was shown to play a role in diabetic glomerulosclerosis after glomerulosclerosis was confirmed to be reversed following pancreatic transplantation in type 1 diabetes [6]. Plasminogen activator inhibitor-1 (PAI-1), a serpin (serine protease inhibitor), is a 50-kDa single-chain glycoprotein that inhibits urokinase plasminogen activator and tissue plasminogen activator, thereby hindering plasminogen cleavage into active plasmin and blocking fibrinolysis [7]. PAI-1 plays a crucial role in several other pathophysiological conditions, including wound healing, obesity, metabolic syndrome, cardiovascular disease, and cancer [7]. Recently, PAI-1 has emerged as a powerful fibrogenic mediator in kidney diseases, including diabetic nephropathy [8, 9] and anti-Thy-1-antibody-mediated glomerulonephritis [10]. PAI-1 overexpression in mice exacerbates kidney fibrosis in obstructive kidney disease, and this is associated with an increase in interstitial macrophage recruitment, interstitial myofibroblast density, and expression of transforming growth factor (TGF)-β1 and collagen I mRNAs [11]. Conversely, PAI-1 deficiency attenuates diabetic nephropathy [12–14], and disruption of the PAI-1 gene markedly attenuates thrombosis and fibrosis in mice [12, 15, 16]. Therefore, inhibition of PAI-1 gene expression might exert critical renoprotective effects [17], and the discovery of specific PAI-1 antagonists might yield new therapeutic approaches [18].

Gene knockout is a powerful technology for screening and demonstration of the suitability of therapeutic targets, but its use in humans is currently limited. Consequently, the use of orally active small-molecule PAI-1 inhibitors (TM5275 and TM5441) could emerge as a practical therapeutic intervention. TM5275 or TM5441 which have been developed with consideration of the three-dimensional structure of PAI-1 [19], have been shown not to inhibit other serpins such as antithrombin III and α2-antiplasmin [20]. TM5275 provides antithrombotic benefits without inducing bleeding episodes in rats and nonhuman primates [19], and it exhibited antifibrotic activity in a murine model of TGF-β-induced lung fibrosis [21]. Recent studies have revealed anti-tumorigenic and anti-angiogenic activity for TM5275 and TM5441 in mice [22], and also that TM5441 inhibits hypertension, cardiac hypertrophy, and vascular fibrosis [20]. However, no report has described the effect of these TM compounds on kidney fibrosis and inflammation in diabetic mice.

Our specific aim in this study was to evaluate the renoprotective effect of the TM compounds TM5275 and TM5441 in diabetes-induced kidney injury. We first examined the *in vivo* effects of the TM compounds on kidney injury in diabetic mice, and then confirmed the effects of the compounds on recombinant PAI-1-induced ECM deposition, monocyte chemotactic protein-1 (MCP-1) expression, and plasmin activity *in vitro*.

## Methods and Materials

### Chemicals and reagents

All chemicals were obtained from Sigma-Aldrich (St. Louis, MO, USA), unless stated otherwise.

### Animals

We used 6-week-old male C57BL/6 mice (Japan SLC Inc., Hamamatsu, Japan), which were divided into 6 groups. Diabetes was induced by intraperitoneally injecting the mice with 150 mg/kg streptozotocin (STZ). Age-matched control mice were injected with an equivalent volume of sodium citrate buffer (100 mM sodium citrate, 100 mM citric acid, pH 4.5). TM5275 at 50 mg/kg/day and TM5441 at 10 mg/kg/day were orally administered in control and diabetic mice for 16 weeks. The effective doses of TM5275 and TM5441 were determined based on previous studies [19, 21, 22] and our preliminary studies (data not shown). Mice that were not administered the TM compounds were injected with an equivalent volume of 0.5% carboxymethyl cellulose, the vehicle for TM5275 and TM5441. Mice were monitored at least once a day, and no deaths occurred during the experimental period. All mice were sacrificed at 16 weeks after STZ injection via anesthesia with 16.5% urethane (10 mL/kg). Blood was collected in a heparinized syringe. We collected blood for measurement of plasma glucose and creatinine, urine for protein measurement, and kidneys for immunohistochemical analysis. All animal experiments were approved by the Institutional Animal Care and Use Committee (ELAGC-09-1014) of Ewha Womans University.

### Cell culture

Mouse proximal tubular epithelial (mProx24) cells were provided by Dr. Takeshi Sugaya (St. Marianna University School of Medicine, Kanagawa, Japan). Cells were cultured in DMEM (Life Technologies, Carlsbad, CA, USA) containing 10% fetal bovine serum, 100 U/mL penicillin, and 100 g/mL streptomycin at 37°C in a humidified 5% CO_2_ atmosphere. Near-confluent cells were incubated with serum-free media for 24 h and pretreated with TM5275 at 50 μM or TM5441 at 10 μM for 4 h before stimulation with recombinant PAI-1 (Calbiochem, La Jolla, CA, USA; approximately 90% biological activity).

### Measurement of blood parameters

Blood samples were collected before the mice were sacrificed. Blood glucose was measured using the glucose oxidase method, and plasma creatinine was measured using a Detect X Serum Creatinine Detection Kit (Arbor Assays, Ann Arbor, MI, USA).

### Measurement of urine parameters

Before mice were sacrificed, urine samples were collected in a metabolic cage for 24 h and centrifuged at 3,000 rpm for 10 min. Urinary proteins in the supernatants were analyzed using the Bradford method and sodium dodecyl sulfate (SDS)-PAGE [23]; all samples were assayed in duplicate. Samples were mixed with sample buffer containing SDS and β-mercaptoethanol and heated at 95°C for 7 min. After electrophoresis, the 10% gels were stained (overnight, with gentle agitation) with Coomassie Brilliant Blue solution (0.2% Coomassie Brilliant Blue R-250 (Bio-Rad Laboratories, Hercules, CA, USA), 50% methanol, and 10% glacial acetic acid) and then destained in a destaining solution (40% methanol and 10% glacial acetic acid), which was replenished several times until the gel background staining was completely eliminated. Bovine serum albumin was used as a control, and an HP4070 Photosmart Scanner was used for imaging; ImageJ software was used for densitometric analysis of the albumin protein band.

### Histology and immunohistochemistry

The right kidney was fixed with 2% paraformaldehyde-lysine-periodate (pH 7.4), dehydrated, embedded in paraffin, and sectioned. Sections were stained with periodic acid–Schiff reagent, and in these sections obtained from each mouse kidney, 20 superficial glomeruli were randomly selected for analysis of glomerular volume and fractional mesangial area (FMA). To examine the collagen matrix, the paraffin-embedded sections were stained using a Masson trichrome stain kit (HT15-1KT) and picrosirius red stain (1:200). For immunohistochemistry, we used anti-fibronectin (1:200) and anti-F4/80 (1:100) antibodies (Santa Cruz Biotechnology, Inc., Santa Cruz, CA, USA). Images were captured using a Zeiss microscope equipped with an Axio Cam HRC digital camera and Axio Cam software (Carl Zeiss, Thornwood, NY, USA), and then quantified using Image-Pro Plus 4.5 software (Media Cybernetics, Silver Springs, MD, USA) as previously described [23].

### Real-time RT-PCR

Total RNA was extracted using TRIzol reagent (Life Technologies), and mRNA expression was measured by means of real-time PCR performed using an ABI7300 system (Applied Biosystems, Carlsbad, CA, USA) and 20-μL reaction volumes containing cDNA transcripts, primer pairs, and SYBR Green PCR Master Mix (Applied Biosystems) as described previously [23]. The primer sequences were as follows: PAI-1: forward, 5'ccttgcttgcctcatcctgg3', reverse, 5'ctggaagagcttgaagaagtgg3' (product, 406 bp); TGF-β1: forward, 5'caggagcgcacaatcatgtt3', reverse, 5'ctttaggaaggacctgggtt3' (258 bp); fibronectin: forward, 5'tgcctcgggaatggaaag3', reverse, 5'atggtagtctccccatcgtcata3' (78 bp); collagen Iα1: forward, 5'cggatagcagattgagaacatccg3', reverse, 5'cggctgagtacggaacaccac3' (201 bp); collagen Iα2: forward, 5'cagaacatcacctaccactgcaa3', reverse: 5'ttcaacatcgttggaaccctg3' (101 bp); α-smooth muscle actin (SMA): forward, 5'gtcccagacatcagggagtaa3', reverse, 5'tcggatacttcagcgtcagga3' (102 bp); MCP-1: forward, 5'-cttctgggcctgctgttca-3', reverse, 5'-ccagcctactcattgggatca-3' (127 bp); and 18S: forward, 5'-cgaaagcatttgccaagaat-3', reverse, 5'-agtcggcatcgtttatggtc-3' (102 bp). We used 18S rRNA as an internal control for normalizing gene expression.

### Plasmin activity

Plasmin activity was determined by using a plasmin-specific fluorogenic substrate: N-succinyl-Ala-Phe-Lys 7-amido-4-methylcoumarin acetate salt. Each reaction was initiated by adding the substrate, after which the contents of the tube were immediately mixed and incubated at 37°C; after the incubation period, the reaction was stopped by adding 25 μL of soybean trypsin inhibitor. The fluorescence of each sample was measured in a fluorometer using 355 and 460 nm as the excitation and emission wavelengths, and a standard curve was prepared using human plasmin (American Diagnostica, Greenwich, CT, USA).

### Statistical analysis

Results are expressed as the mean ± SE, and the mean values obtained from each group were compared by ANOVA with subsequent Fisher’s least significant difference test. Non-parametric analyses were also used where appropriate. p < 0.05 was considered statistically significant.

## Results

### TM compounds improve kidney function and morphology in STZ-induced diabetic mice

**Fig 1:**
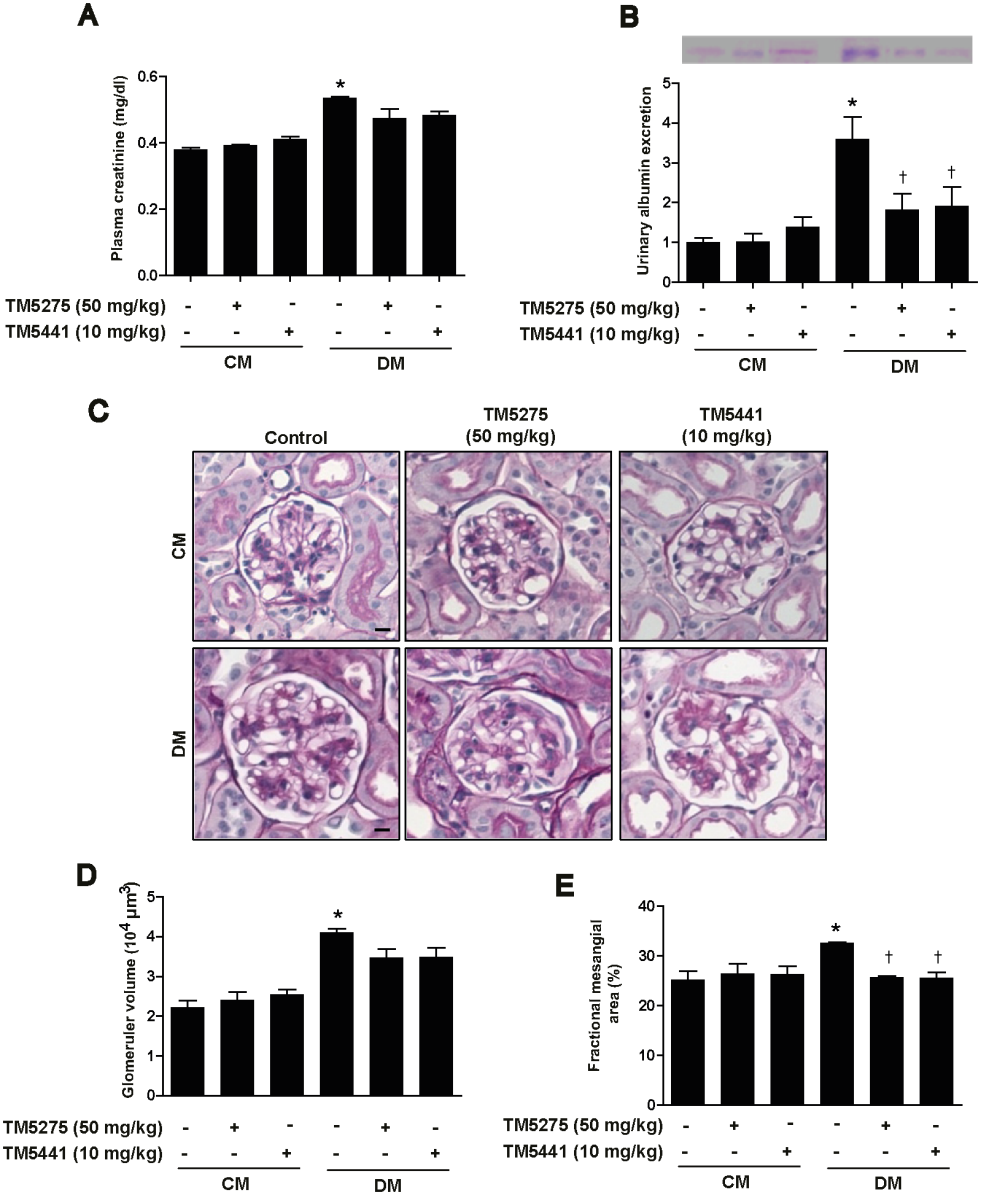
TM compounds improve kidney function and morphology in STZ-induced diabetic mice. Diabetes was induced in mice by intraperitoneally injecting STZ (150 mg/kg), and then either TM5275 (50 mg/kg/day) or TM5441 (10 mg/kg/day) was administered orally for 16 weeks to the diabetic and age-matched control mice. After 16 weeks, blood was collected for analysis of (A) plasma creatinine, and urine was collected for (B) protein analysis by using the Bradford assay and SDS-PAGE. (C) Kidneys were fixed in paraffin and cut into 4-μm sections that were subsequently stained with PAS (periodic acid–Schiff) reagent. Scale bar: 10 μm; original magnification: 630×. After PAS staining, (D) glomerular volume and (E) FMA were analyzed using Image-Pro Plus 4.5.1. CM, control mice; DM, STZ-induced diabetic mice. Data are presented as the mean ± SE of 5–8 mice/group; *p < 0.05 vs. CM, †p < 0.05 vs. DM.

At 16 weeks after STZ injection, mice showed lower body weight gain and increased plasma glucose level as compared to age-matched control mice. The plasma glucose level in diabetic mice was not affected by treatment with either TM5275 (50 mg/kg) or TM5441 (10 mg/kg) (Table 1). The diabetic mice also showed increased kidney-to-bodyweight ratio (Table 1) and plasma creatinine level (Fig 1A), and these alterations were again not markedly affected by the TM compounds (Fig 1A). Furthermore, STZ-induced diabetic mice showed a significant increase in urinary albumin excretion, glomerular volume, and FMA. Intriguingly, the TM compounds effectively reduced albuminuria and FMA in the diabetic mice (Fig 1B, 1C and 1E), although neither inhibitor exerted a large effect on STZ-induced glomerular hypertrophy (Fig 1C and 1D). Together, these data demonstrate that the TM compounds TM5275 and TM5441 protect mice against diabetes-induced albuminuria and mesangial expansion without affecting hyperglycemia.

**Table 1:** Metabolic characteristics of STZ-induced diabetic mice after treatment with PAI-1 inhibitors.

	Control mice			Diabetic mice		
**TM5275 (50 mg/kg)**	–	+	–	–	+	–
**TM5441 (10 mg/kg)**	–	–	+	–	–	+
**Body weight (g)**	28 ± 0.7	29 ± 0.6	28 ± 1.1	23 ± 0.4*	23 ± 0.8*	22 ± 0.6*
**Kidney weight /body weight (g/kg)**	6.1 ± 0.9	5.9 ± 0.4	5.6 ± 0.4	9.2 ± 1.2*	9.3 ± 1.4*	10.1 ± 0.6*
**Plasma glucose (mg/dL)**	204 ± 12	153 ± 12	167 ± 9	427 ± 67*	374 ± 53*	510 ± 53*

Diabetes was induced through intraperitoneal injection of STZ (150 mg/kg), and then the mice were treated with TM5275 or TM5441 for 16 weeks. At 16 weeks after STZ injection, all mice were sacrificed and blood was collected for measurement of plasma glucose. Data are presented as the mean ± SE of 5–8 mice/group;

*p < 0.05 vs. control mice.

### TM compounds inhibit kidney fibrosis in STZ-induced diabetic mice

**Fig 2:**
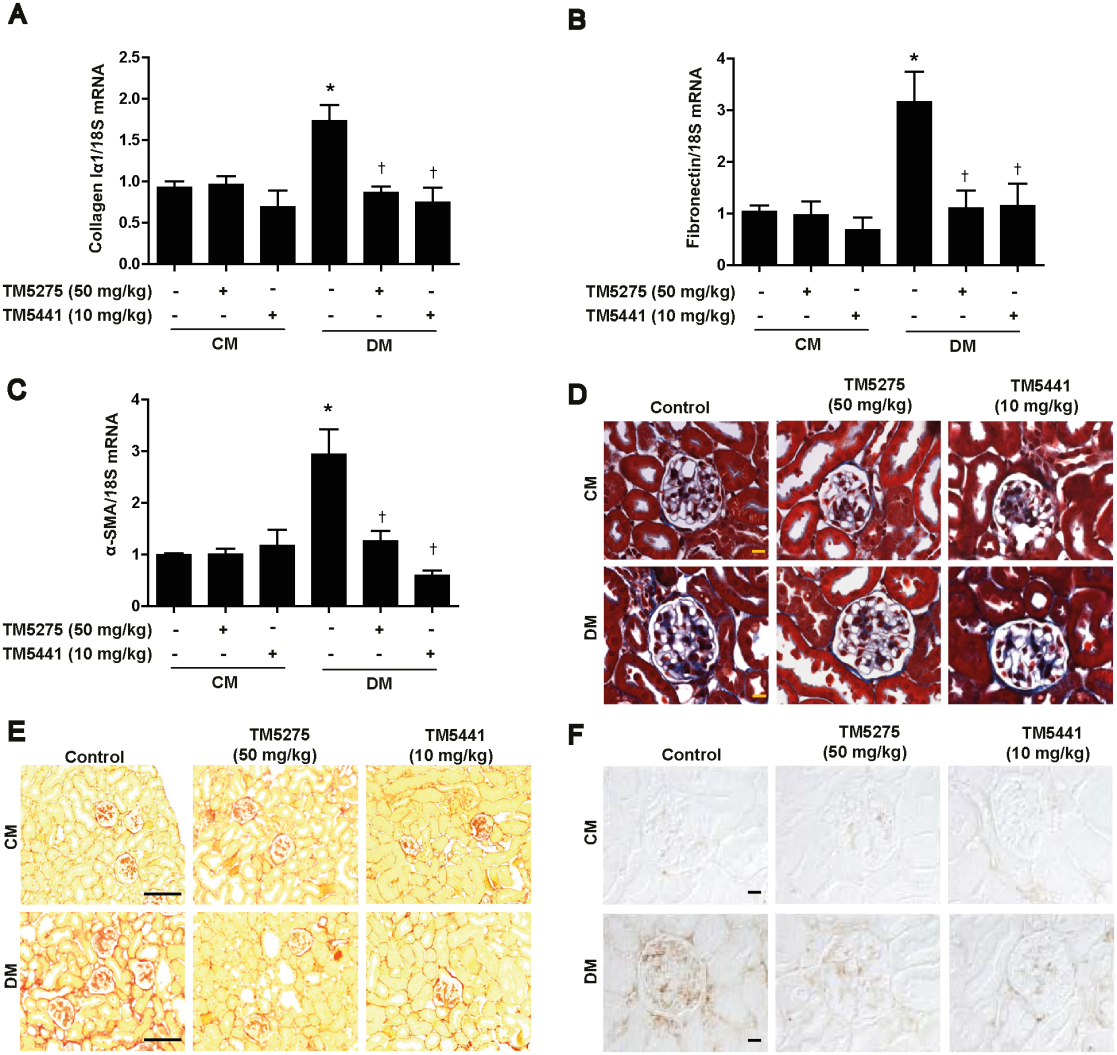
TM compounds inhibit kidney fibrosis in STZ-induced diabetic mice. After diabetic mice were treated with TM compounds for 16 weeks, the mRNA and protein levels of fibrosis markers in kidney tissue were measured. (A) Collagen Iα1, (B) fibronectin, and (C) α-SMA mRNA expression levels were measured using real-time PCR. Paraffin-embedded kidney sections were stained with (D) Masson trichrome stain (original magnification: 630×; scale bar: 10 μm), (E) picrosirius red (original magnification: 200×; scale bar: 100 μm), or anti-fibronectin antibodies; original magnification: 630×; scale bar: 10 μm). CM, control mice; DM, STZ-induced diabetic mice. Data are presented as the mean ± SE of 5–8 mice/group; *p < 0.05 vs CM, †p < 0.05 vs DM.

Next, we measured fibrosis indices because fibrosis is the most critical pathological process underlying the progression of diabetic kidney disease [24]. Our results showed that treatment with the TM compounds inhibited STZ-induced upregulation of collagen I (Fig 2A), fibronectin (Fig 2B), and α-SMA (Fig 2C) mRNAs in diabetic kidneys. To further confirm the effects of the TM compounds on kidney fibrosis, we stained paraffin-embedded kidney sections with Masson’s modified trichrome, picrosirius red, and anti-fibronectin antibodies. Administration of TM compounds effectively inhibited STZ-induced collagen accumulation (Fig 2D and 2E) and fibronectin expression (Fig 2F) in diabetic kidneys. PAI-1 protein expression was also increased in the tubules of STZ-induced diabetic kidneys, which was effectively inhibited by TM compounds (S1 Fig). It is notable that PAI-1 mRNA expression was upregulated in cultured tubular epithelial as well as mesangial cells in response to palmitate (a model of diabetic stress) as shown in S1 Fig. These results demonstrate that the TM compounds protect mice against kidney fibrosis in the early stage of diabetes.

### TM compounds inhibit kidney inflammation in STZ-induced diabetic mice

**Fig 3:**
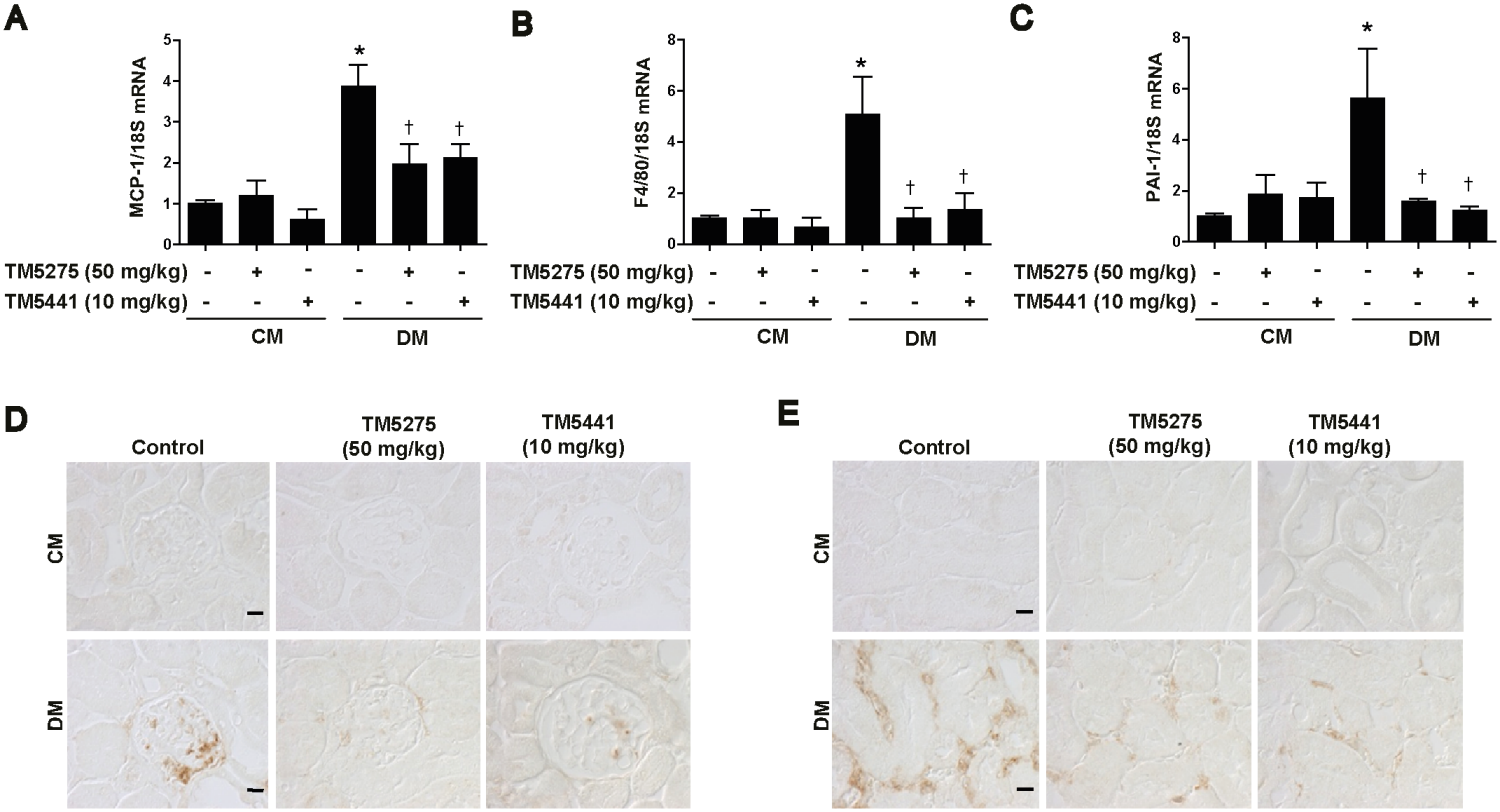
TM compounds inhibit kidney inflammation in STZ-induced diabetic mice. After diabetic mice were treated for 16 weeks with TM compounds, mRNA and protein expression levels of inflammatory cytokines were measured in the kidney tissue. Real-time PCR analysis of the mRNA expression of (A) MCP-1, (B) F4/80, and (C) PAI-1. (D, E) Paraffin-embedded kidney sections were stained with anti-F4/80 antibodies (1:200; original magnification: 630×; scale bar: 10 μm). CM, control mice; DM, STZ-induced diabetic mice. Data are presented as the mean ± SE of 5–8 mice/group; *p < 0.05 vs CM, †p < 0.05 vs DM.

Macrophages are the major component in inflammation. Therefore, to examine the effects of TM compounds on macrophage infiltration induced by STZ in the kidney, we analyzed the mRNA levels of MCP-1 and F4/80 in kidney tissues by using real-time RT-PCR. STZ-induced mRNA expression of MCP-1 and F4/80 was significantly inhibited by treatment with the TM compounds (Fig 3A and 3B). Interestingly, administration of these compounds also effectively reduced STZ-induced PAI-1 mRNA levels in the mouse kidney (Fig 3C). To further confirm the effect of TM compounds on macrophage infiltration, paraffin-embedded kidney sections were stained with anti-F4/80 antibodies. Macrophage accumulation in kidney glomeruli and tubules was higher in the STZ-induced diabetic mice than in control mice (Fig 3D and 3E) but decreased following treatment with the TM compounds (Fig 3D and 3E). Collectively, these results and the mRNA expression profile of macrophage infiltration marker genes described above demonstrate that renal inflammation induced by macrophage infiltration in the kidney of STZ-induced diabetic mice is effectively prevented by TM compounds.

### TM compounds inhibit PAI-1-induced fibrotic and inflammatory responses *in vitro*

**Fig 4:**
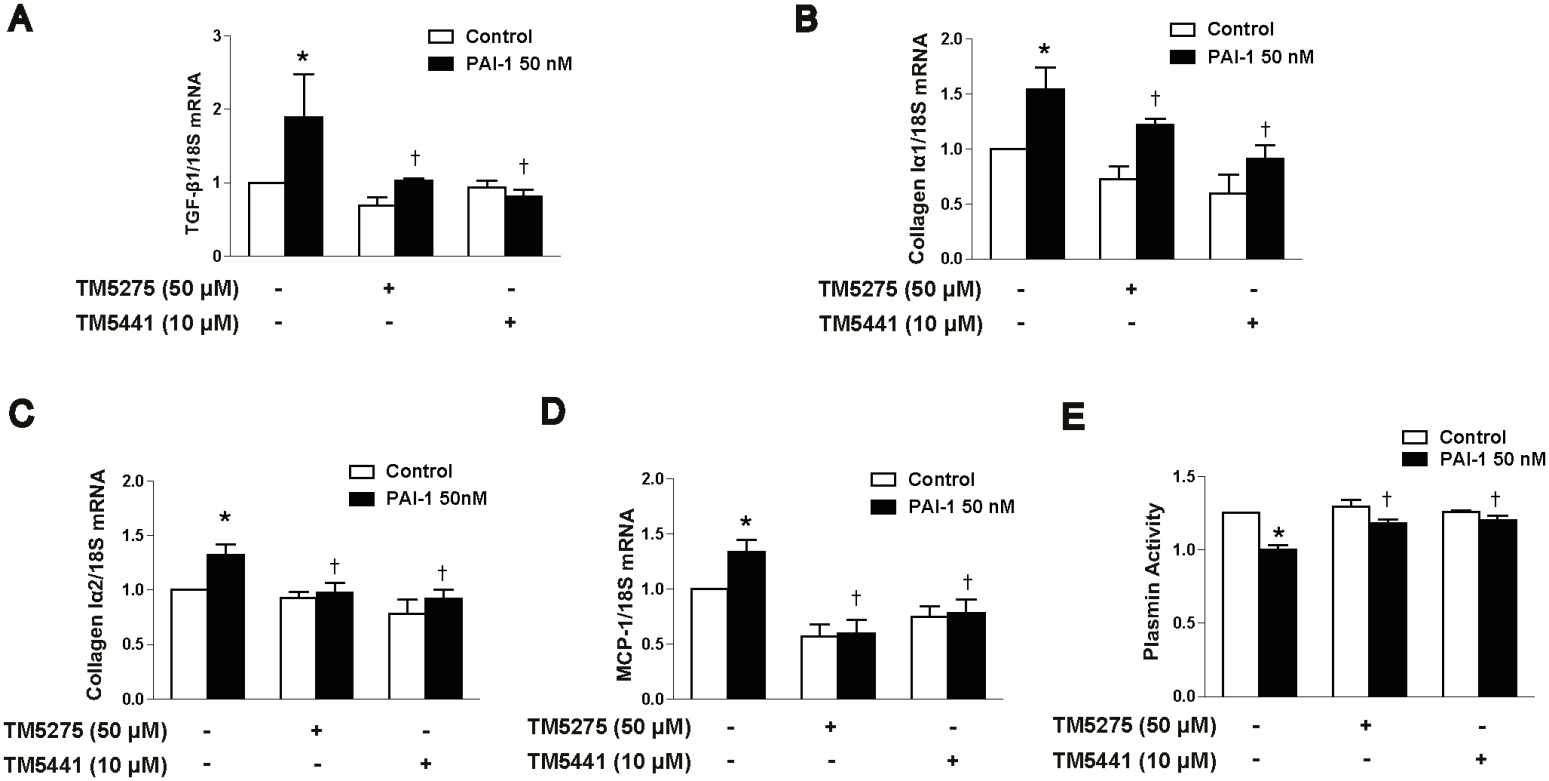
TM compounds inhibit PAI-1-induced fibrotic and inflammatory responses *in vitro*. We treated mProx cells with TM compounds for 4 h and then stimulated them with 50 nM recombinant PAI-1 for 24 h. Real-time RT-PCR was used to measure the mRNA expression of (A) TGF-β1, (B) collagen Iα1, (C) collagen Iα2, and (D) MCP-1. (E) Plasmin activity was also measured. Data are presented as the mean ± SE of 4 experiments; *p < 0.05 vs control, †p < 0.05 vs PAI-1.

To confirm the efficacy of TM compounds as PAI-1 inhibitors in the kidney, we investigated the effect of TM5275 and TM5441 on PAI-1-induced markers of both fibrosis and inflammation in mProx cells. PAI-1 treatment significantly increased the mRNA expression of TGF-β, collagen Iα1, collagen Iα2, and MCP-1 (Fig 4A–4D), which suggests that PAI-1 exerted profibrotic and proinflammatory effects; notably, treatment with the TM compounds effectively decreased the PAI-1-induced fibrotic and inflammatory responses (Fig 4A–4D), which confirms the effectiveness of the TM compounds as PAI-1 inhibitors. As expected, PAI-1-induced suppression of plasmin activity was also inhibited following treatment with the TM compounds (Fig 4E). Together, these results suggest that TM compounds can effectively improve PAI-1-induced fibrotic and inflammatory responses in the kidney.

## Discussion

This study was conducted to provide experimental evidence that two novel orally active PAI-1 inhibitors, TM5275 and TM5441, can prevent the development and progression of diabetic kidney injury, and to suggest the use of TM compounds as a new strategy for preventing diabetic nephropathy. Accordingly, at 16 weeks after injection of STZ, the diabetic mice showed an increase (relative to control mice) in various parameters of kidney injury, such as plasma creatinine level, urinary albumin excretion, kidney-to-body weight ratio, glomerular volume, and FMA. Notably, treatment with TM5275 and TM5441 effectively reduced urinary albumin excretion and FMA. Consistent with our results, another compound of TM series significantly reduced proteinuria in NEP25/LMB2 podocyte injury mouse model [25]. With regard to the mechanic explanation for the role of PAI-1 on urine albumin levels, PAI-1/uPA complex-mediated uPAR-dependent podocyte β1-integrin endocytosis has been proposed in progressive podocyte injury leading to proteinuria [25]. However, inhibition of PAI-1 did not affect plasma glucose levels in STZ-induced diabetic mice, which agreed with the results of a previous study [26]. These data indicate that TM compounds improve kidney function and morphology in diabetic mice.

TM5007, the parent compound of TM5275 and TM5441, prevents bleomycin-induced lung fibrosis in mice [27], and tiplaxtinin (an indole oxoacetic acid derivative) attenuates angiotensin II-induced aortic remodeling in mice [16]. These two previous studies suggest that the best-in-class PAI-1 inhibitors could be effective antifibrotic agents. Here, our study demonstrating the antifibrotic effect of TM5275 and TM5441 in diabetic kidney injury is consistent with previous studies conducted using PAI-1 null mice. PAI-1 deficiency reduces ECM accumulation and tubulointerstitial or glomerular fibrosis in STZ-induced diabetic mice [12, 14] and *db/db* diabetic mice [28]. PAI-1 deficiency was also shown to reduce fibrosis [13] and collagen accumulation in a model of obstructive nephropathy [29]. Conversely, PAI-1 overexpression exacerbates fibrosis in obstructed kidneys [11]. In this study, TM compounds reduced the upregulation of collagen I, fibronectin, and PAI-1 mRNA in the kidneys of STZ-induced diabetic mice, which indicates that PAI-1 might induce ECM accumulation by increasing the mRNA expression of each of these ECM components, and that the fibrotic effect of PAI-1 is partly caused by a mechanism that is independent of its action on enzymatic conversion of plasminogen to plasmin. Furthermore, our results confirmed the *in vitro* effects of the TM compounds: both compounds effectively inhibited PAI-1-induced collagen I and TGF-β mRNA expression in cultured kidney tubular epithelial cells. In the line with our results, knockout of the PAI-1 gene suppresses the expression of high glucose-induced TGF-β1 mRNA, whereas recombinant PAI-1 restores the inducibility of TGF-β1 by high glucose in PAI-1 knockout kidney cells [14]. In addition, PAI-1 transgenic mice show higher expression of TGF-β1 mRNA in response to unilateral ureteral obstruction [11]. Altogether, these data strongly support the notion that PAI-1 positively regulates TGF-β1 gene expression during renal fibrosis in diabetes. Receptor-dependent signal transduction of PAI-1 has also been previously reported in mesangial cells [12, 14].

Another possible mechanism of action of the TM compounds is anti-inflammation. Previous studies have reported a relationship between inflammation and PAI-1: PAI-1 exhibits monocyte chemoattractant properties, and this depends on the expression of LDL receptor-associated protein (LRP) [8, 30]. The interaction of PAI-1 with LRP directs the migration of monocytes, vascular smooth muscle cells, and fibroblasts [17]. PAI-1-deficient mice with obstructive nephropathy showed a substantial delay in the recruitment of macrophages and myofibroblasts to the interstitium [13], and macrophage infiltration was also decreased in a mutant, non-inhibitory PAI-1-treated experimental glomerulonephritis model [31]. Furthermore, in PAI-1 transgenic mice, severe fibrosis in the obstructed kidneys was associated with intense recruitment of interstitial macrophages [11]. Given the role of macrophage infiltration in kidney fibrosis [32], we measured macrophage infiltration and found that it was increased in the kidneys of diabetic mice and that treatment with the TM compounds markedly inhibited this infiltration, as indicated by MCP-1 and F4/80 mRNA levels and F4/80 immunohistochemistry. Moreover, recombinant PAI-1-induced MCP-1 mRNA upregulation was reversed following treatment with the TM compounds.

Interestingly, treatment with the TM compounds also effectively reduced PAI-1 mRNA and protein levels in the kidneys of diabetic mice. Suppression of PAI-I mRNAs in experimental animals after administration of PAI-1 inhibitors have been also demonstrated previously in other kidney disease models, such as the anti-Thy-1 rat glomerulonephritis model [33] as well as in the rodent multiple sclerosis model [34]. Altogether, these results suggest the positive feedback loop between PAI-1 activity and expression.

A few questions related to the present findings remain to be answered. For instance, limitations of STZ-induced diabetes in C57BL/6 mice as a model for human diabetic nephropathy have been reported [35]. Therefore, the effects of the TM compounds must be confirmed in eNOS-deficient diabetic mice [35]. Another key question is whether or not the TM compounds will be therapeutically effective if administered at later time points when kidney injury has already been established.

In summary, the TM compounds improved kidney function, fibrosis, and inflammation in STZ-induced diabetic mice. Therefore, oral administration of TM5275 and TM5441, two novel PAI-1 inhibitors that do not induce bleeding episodes, could emerge as an effective measure for treating diabetic nephropathy.

## Supporting Information

S1 Fig TM compounds inhibit diabetes-induced PAI-1 upregulation. (A) STZ-induced diabetic mice were orally administered with TM5275 (50 mg/kg/day) or TM5441 (10 mg/kg/day) for 16 weeks. Paraffin-embedded kidney sections were stained with anti-PAI-1 antibodies (1:200, Santa Cruz Biotechnology, Inc., Santa Cruz, CA, USA); original magnification: 200×; scale bar: 50 μm. CM, control mice; DM, STZ-induced diabetic mice and representative image has been shown. (B) mProx cells and (C) mesangial cells were treated with palmitate (400 μM) for 10 h. Real-time RT-PCR was used to measure the mRNA expression of PAI-1. Data are presented as the mean ± SE of 4 experiments; *p < 0.05 vs control, BSA was used as control.
(TIF)

S1 Materials and Methods Palmitic acid preparation and cell culture. Palmitic acid was dissolved in 50% ethanol and heated to 60°C to obtain a clear solution. Fatty acid free-BSA was dissolved in PBS. The dissolved palmitic acid solution was added little by little in warmed 10% BSA (45~52°C). Finally, pH of the combined solution was adjusted to 7.0~7.4 by adding NaOH slowly, and aliquots were frozen and stored at -20°C. In addition to mProx cells (as described in the main text), murine mesangial cells (MES-13, cloned from mice transgenic for the early region of SV-40 virus, passage 25 which was obtained from American Type Culture Collection, Rockville, MD) were used. Mesangial cells were cultured in DMEM containing 5% fetal bovine serum (FBS; Life Technologies BRL, Gaitherburg, MD), 100 U/ml penicillin, 100 g/ml streptomycin, 44 mM NaHCO_3_, and 14 mM N-hydroxy-ethylpiperazine-N'-2-ethane sulfonic acid (HEPES). Near-confluent mesangial cells were incubated with serum-free media for 24 h to arrest and synchronize the cell growth. After this time period, the media were changed to fresh serum-free DMEM and cells were stimulated with 400 μM palmitate for 10 h.
(DOCX)
