# Supplementary figures and images for: Novel Plasminogen Activator Inhibitor-1 Inhibitors Prevent Diabetic Kidney Injury in a Mouse Model
Source: PLoS One. 2016 Jun 3;11(6):e0157012. doi: 10.1371/journal.pone.0157012 (PMC4892642; doi:10.1371/journal.pone.0157012)

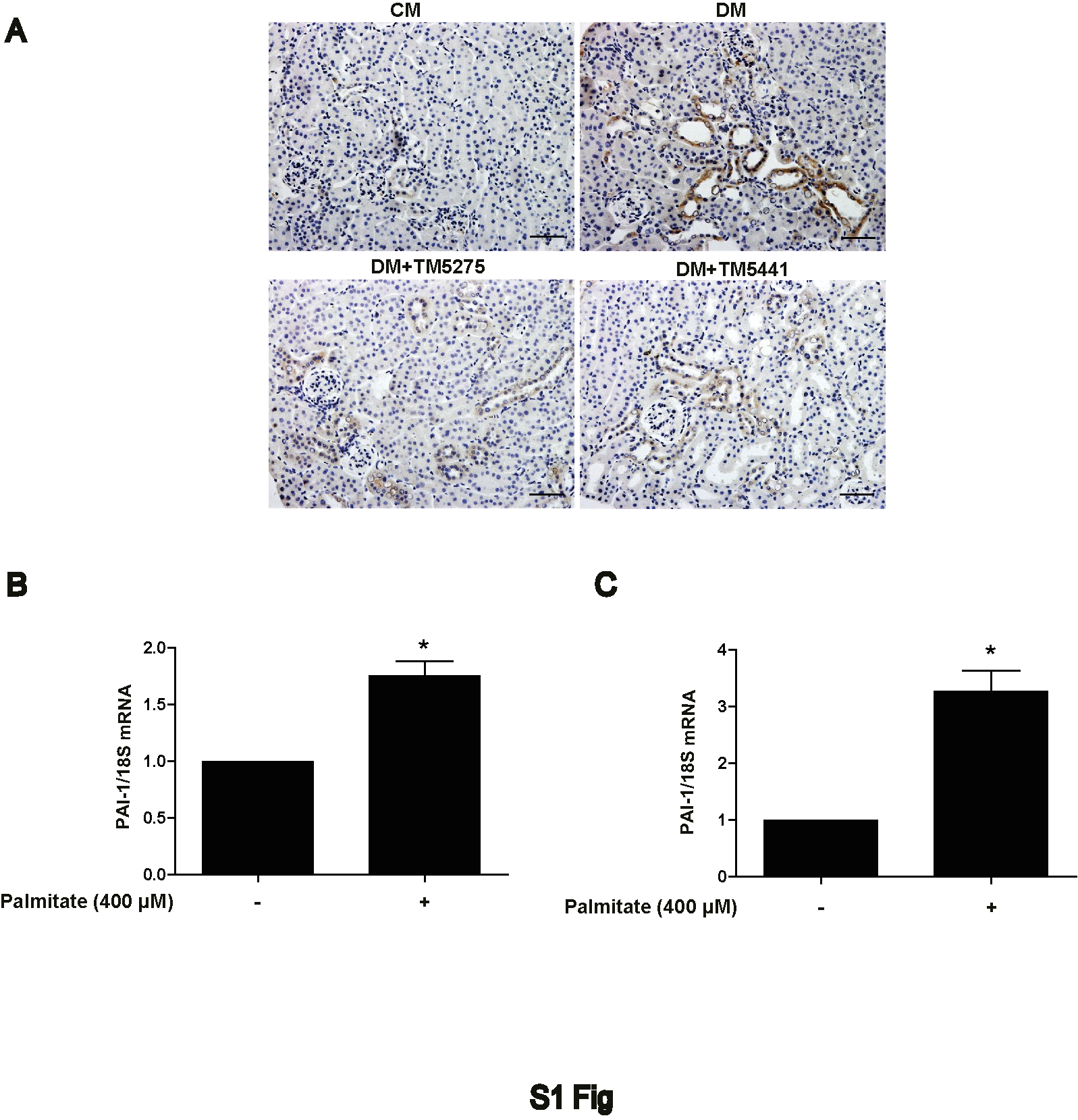

Supplement: S1 Fig — (A) STZ-induced diabetic mice were orally administered with TM5275 (50 mg/kg/day) or TM5441 (10 mg/kg/day) for 16 weeks. Paraffin-embedded kidney sections were stained with anti-PAI-1 antibodies (1:200, Santa Cruz Biotechnology, Inc., Santa Cruz, CA, USA); original magnification: 200×; scale bar: 50 μm. CM, control mice; DM, STZ-induced diabetic mice and representative image has been shown. (B) mProx cells and (C) mesangial cells were treated with palmitate (400 μM) for 10 h. Real-time RT-PCR was used to measure the mRNA expression of PAI-1. Data are presented as the mean ± SE of 4 experiments; *p < 0.05 vs control, BSA was used as control. (TIF) [file pone.0157012.s001.tif]
