# Supplementary material for: Novel Plasminogen Activator Inhibitor-1 Inhibitors Prevent Diabetic Kidney Injury in a Mouse Model
Source: PLoS One. 2016 Jun 3;11(6):e0157012. doi: 10.1371/journal.pone.0157012 (PMC4892642; doi:10.1371/journal.pone.0157012)
Supplement: S1 Materials and Methods — Palmitic acid was dissolved in 50% ethanol and heated to 60°C to obtain a clear solution. Fatty acid free-BSA was dissolved in PBS. The dissolved palmitic acid solution was added little by little in warmed 10% BSA (45~52°C). Finally, pH of the combined solution was adjusted to 7.0~7.4 by adding NaOH slowly, and aliquots were frozen and stored at -20°C. In addition to mProx cells (as described in the main text), murine mesangial cells (MES-13, cloned from mice transgenic for the early region of SV-40 virus, passage 25 which was obtained from American Type Culture Collection, Rockville, MD) were used. Mesangial cells were cultured in DMEM containing 5% fetal bovine serum (FBS; Life Technologies BRL, Gaitherburg, MD), 100 U/ml penicillin, 100 g/ml streptomycin, 44 mM NaHCO3, and 14 mM N-hydroxy-ethylpiperazine-N'-2-ethane sulfonic acid (HEPES). Near-confluent mesangial cells were incubated with serum-free media for 24 h to arrest and synchronize the cell growth. After this time period, the media were changed to fresh serum-free DMEM and cells were stimulated with 400 μM palmitate for 10 h. (DOCX) [file pone.0157012.s002.docx]

**Supporting Information**

**S1 Materials and Methods. Palmitic acid preparation and cell culture.** Palmitic acid was dissolved in 50% ethanol and heated to 60°C to obtain a clear solution. Fatty acid free-BSA was dissolved in PBS. The dissolved palmitic acid solution was added little by little in warmed 10% BSA (45~52°C). Finally, pH of the combined solution was adjusted to 7.0~7.4 by adding NaOH slowly, and aliquots were frozen and stored at -20°C.

In addition to mProx cells (as described in the main text), murine mesangial cells (MES-13, cloned from mice transgenic for the early region of SV-40 virus, passage 25 which was obtained from American Type Culture Collection, Rockville, MD) were used. Mesangial cells were cultured in DMEM containing 5% fetal bovine serum (FBS; Life Technologies BRL, Gaitherburg, MD), 100 U/ml penicillin, 100 g/ml streptomycin, 44 mM NaHCO_3_, and 14 mM N-hydroxy-ethylpiperazine-N'-2-ethane sulfonic acid (HEPES). Near-confluent mesangial cells were incubated with serum-free media for 24 h to arrest and synchronize the cell growth. After this time period, the media were changed to fresh serum-free DMEM and cells were stimulated with 400 µM palmitate for 10 h.

**S1 Fig. TM compounds inhibit diabetes-induced PAI-1 upregulation. (**A) STZ-induced diabetic mice were orally administered with TM5275 (50 mg/kg/day) or TM5441 (10 mg/kg/day) for 16 weeks. Paraffin-embedded kidney sections were stained with anti-PAI-1 antibodies (1:200, Santa Cruz Biotechnology, Inc., Santa Cruz, CA, USA); original magnification: 200×; scale bar: 50 µm. CM, control mice; DM, STZ-induced diabetic mice and representative image has been shown. (B) mProx cells and (C) mesangial cells were treated with palmitate (400 µM) for 10 h. Real-time RT-PCR was used to measure the mRNA expression of PAI-1. Data are presented as the mean ± SE of 4 experiments; *p < 0.05 vs control, BSA was used as control.
